# Composition of cometary particles collected during two periods of the Rosetta mission: multivariate evaluation of mass spectral data

**DOI:** 10.1002/cem.3218

**Published:** 2020-01-27

**Authors:** Kurt Varmuza, Peter Filzmoser, Nicolas Fray, Hervé Cottin, Sihane Merouane, Oliver Stenzel, John Paquette, Jochen Kissel, Christelle Briois, Donia Baklouti, Anaïs Bardyn, Sandra Siljeström, Johan Silén, Martin Hilchenbach

**Affiliations:** ^1^ Institute of Statistics and Mathematical Methods in Economics Vienna University of Technology Vienna Austria; ^2^ LISA, UMR CNRS 7583, Université Paris‐Est‐Créteil, Université de Paris, Institut Pierre Simon Laplace Créteil France; ^3^ Max‐Planck‐Institute for Solar System Research Göttingen Germany; ^4^ Laboratoire de Physique et Chimie de l'Environnement et de l'Espace Université d'Orléans et du CNES Orléans France; ^5^ Institut d'Astrophysique Spatiale Université Paris Sud Orsay France; ^6^ Department of Geology University of Maryland College Park MD USA; ^7^ Bioscience and Materials/Chemistry and Materials RISE Research Institutes of Sweden Stockholm Sweden; ^8^ Finnish Meteorological Institute Helsinki Finland

**Keywords:** comet 67P/Churyumov‐Gerasimenko, KNN classification, random forest classification, time‐of‐flight secondary ion mass spectrometry, variable importance

## Abstract

The instrument COSIMA (COmetary Secondary Ion Mass Analyzer) onboard of the European Space Agency mission Rosetta collected and analyzed dust particles in the neighborhood of comet 67P/Churyumov‐Gerasimenko. The chemical composition of the particle surfaces was characterized by time‐of‐flight secondary ion mass spectrometry. A set of 2213 spectra has been selected, and relative abundances for CH‐containing positive ions as well as positive elemental ions define a set of multivariate data with nine variables. Evaluation by complementary chemometric techniques shows different compositions of sample groups collected during two periods of the mission. The first period was August to November 2014 (far from the Sun); the second period was January 2015 to February 2016 (nearer to the Sun). The applied data evaluation methods consider the compositional nature of the mass spectral data and comprise robust principal component analysis as well as classification with discriminant partial least squares regression, *k*‐nearest neighbor search, and random forest decision trees. The results indicate a high importance of the relative abundances of the secondary ions C^+^ and Fe^+^ for the group separation and demonstrate an enhanced content of carbon‐containing substances in samples collected in the period with smaller distances to the Sun.

## Abstract 2

Extraterrestrial dust particles from the neighborhood of comet 67P/Churyumov‐Gerasimenko have been collected by the instrument COSIMA onboard of the European Space Agency mission Rosetta. The composition of the particles was characterized by time‐of‐flight secondary ion mass spectrometry giving evidence of minerals and macromolecular organic substances. A specific multivariate evaluation of the mass spectral data by chemometric methods indicates an enhanced content of carbon‐containing substances in samples collected in a period with smaller distances to the Sun.

## 1 INTRODUCTION

The comet 67P/Churyumov‐Gerasimenko (short *67P*) was explored and continuously observed in situ between August 2014 and September 2016 by instruments onboard the spacecraft Rosetta, launched by the European Space Agency on 2 March 2004.^1^ The distance between Rosetta and the comet (nucleus size 6 km × 4 km × 3 km) was typically between 10 and 150 km. The heliocentric distance between the comet and the Sun during this time was between 1.2 and 3.8 AU (1 AU, astronomical unit; defined as 149 597 870 700 m; approximately the mean distance between Earth and Sun). The activity of the comet in terms of emitted gas and solid particles is highly dependent on the heliocentric distance. The aim of this work was to search for potential differences in the chemical composition of cometary particles collected during two periods of the mission.

The instrument COSIMA (COmetary Secondary Ions Mass Analyzer)^2^ onboard of Rosetta collected cometary particles on metal targets (1 cm × 1 cm, with a porous gold surface) during exposures outside the instrument for typically 1 day to 1 week. A built‐in microscope^3^ made images from the targets with a resolution of 14 μm and 1024 × 1024 pixel. In total, about 1400 particles (corresponding to about 30 000 particle fragments) have been documented from the obtained images.^4^ The typical particle diameter is 50 to 700 μm; the particle areas have a median of 390 μm^2^ and a maximum of 0.5 mm^2^. The built‐in time‐of‐flight secondary ion mass spectrometer (TOF‐SIMS) measured mass spectra at selected positions on the targets.^5^ The footprint of the primary ion beam was about 35 μm × 50 μm; the mass resolution *m*/Δ*m* was about 500 at mass 12 (C^+^) and about 800 at mass 56 (Fe^+^); Δ*m* is the full width at half maximum peak height. This mass resolution allows a separation of elemental ions from H‐rich carbon containing ions of the same nominal (integer) mass in this mass range. More details of the data collection for the COSIMA instrument are presented in Merouane et al.^4^

More than 33 000 mass spectra have been acquired. Characteristics about the chemical composition of the particles have been derived from these data as follows: the organic substance of the particles is macromolecular,^6^ however, no specific organic compounds could be identified; atomic ratios were estimated as C/Si ~5,^7^ C/H ~1,^8^ and C/N ~30^9^; the presence of the ions C_3_H_0‐4_^+^ and C_4_^+^ in the spectra, as well as the C/H elemental ratios, indicates unsaturated compounds^10^; the elemental composition related to Fe is close to chondritic meteorites but enriched in Li, C, Na, Si, S, K, and Cu and depleted in Mg and Ca.^7^, ^11^ Related to this work are comparisons of the atomic C/Si ratios of particles collected at different heliocentric distances, however, with no clear correlations found between C/Si and the distance from comet to Sun.^7^

The strategy applied in this study for investigating potential differences in the chemical composition of cometary particles is summarized in the following seven items—with details given in the following sections. (a) A set of *n* = 2213 spectra has been selected by automatic procedures applying criteria for the experimental and spectral quality,^7^, ^9^, ^10^, ^11^, ^12^ such as a maximum allowed contamination, reaching minimum ion counts, and the availability of heliocentric distance data. (b) For the *m* = 9 ions of type C_x_H_y_^+^ (for organics) and Mg^+^ and Fe^+^ (for minerals) the ion count data (mass spectral peak heights) were extracted from the mass calibrated raw data, resulting in a set of multivariate data given by a matrix ***X***(*n* × *m*). (c) The *n* spectra were divided into two classes, class 1 for spectra from samples collected at the beginning of the mission near the comet and approaching the Sun with heliocentric distances between 3.57 and 2.93 AU, and class 2 for spectra from particles collected at heliocentric distances between 2.48 and 1.24 AU in the time before and after perihelion (closest distance to sun, 1.24 AU). (d) A robust principal component analysis (PCA) gives an insight into the separation of the two classes. (e) Results from linear and nonlinear classification methods characterize the separability of the two classes. A good separation indicates different chemical compositions as reflected by the used mass spectral data. (f) Variables with high importance for the class separation indicate the specific secondary ions, which are characteristic for the separation. (g) Comparison of the distributions of ion counts and ion count ratios support a preliminary interpretation of different compositions of the particle classes.

## 2 DATA AND METHODS

### 2.1 Spectral data

For this work, SIMS spectra with positive secondary ions have been used. The mass scale of each spectrum has been individually calibrated using the reference ions ^12^C^+^, ^23^Na^+^, and ^28^Si(^12^CH_3_)_3_^+^ by fitting Gauss peaks to the experimental ion count data.^10^ The *m* = 9 ion species considered are C^+^, CH^+^, CH_2_^+^, CH_3_^+^, C_2_H_3_^+^, C_3_H_3_^+^, C_3_H_4_^+^, ^24^Mg^+^, and ^56^Fe^+^. This selection is guided by (a) including ions characteristic for the macromolecular organic material,^6^ as well as for the inorganic material, probably mainly silicates^11^; (b) using ion species that are well separated from others at the same nominal mass; and (c) not using ion species with uncertain contributions from the background (e.g., Na^+^ and Si^+^). Mass spectral peak heights were calculated as the sum of the ion counts within defined mass intervals,^10^ for instance, mass interval 11.960 to 12.030 for ions C^+^ (exact mass 12.0000) or 55.880 to 55.980 for ions Fe^+^ (exact mass 55.9349).

The spectral data show a contamination of the samples by PDMS (polydimethylsiloxane), a common background in SIMS experiments.^7^ The contribution of PDMS to the C_x_H_y_^+^ signals has been approximately subtracted by using peak height ratios from a reference spectrum based on the signal at mass 73 from (CH_3_)_3_Si^+.^ 10 Contributions of unidentified contaminants are considered as part of a chemical noise.

The COSIMA documentation of the collected particles^4^ contains more than 34 000 entries with the *x*‐ and *y*‐coordinates of the particle centers, together with data about the collection period. Mass spectra have been selected if the *x*‐ and *y*‐coordinates of the SIMS measurement are within ±70 μm of the center of a documented particle having a minimum area of 500 μm^2^.

The final data comprise *n* = 2213 spectra with ion count data for *m* = 9 species, constituting matrix ***X***(*n* × *m*). The original ion counts are between 0 and about 10 000 with a median of 155, and a mean of 485. A cutoff for the heliocentric distance of 2.6 AU has been defined, dividing the samples into class 1 with *n*_1_ = 839 spectra from samples collected rather far from the Sun in the time between begin of collections and approaching a distance of about 2.6 AU; class 2 contains *n*_2_ = 1374 spectra from samples collected rather near the Sun at heliocentric distances smaller than 2.6 AU (Table 1). The cutoff of 2.6 AU has been chosen because of a gap in the data between 2.5 and 2.9 AU and because of giving similar class sizes. The range of particle size (measured by the particle area in the images) is similar in both classes with an interquartile range of 1600 to 10 600 μm^2^ for class 1 (first period, far Sun) and 2200 to 8800 μm^2^ for class 2 (second period, near Sun); median and maximum of the particle areas are larger in class 2 than in class 1.

**Table 1:** Class characteristics

Data	Class 1	Class 2
	Period 1, Far Sun	Period 2, Near Sun
Heliocentric distance (AU)	2.93‐3.57	1.24‐2.48
First date of collection begin	August 11, 2014	January 24, 2015
Last date of collection begin	November 21, 2014	February 29, 2016
Number of collection periods	10	13
Number of particles	69	157
Particle size (area in image)		
First to third quartiles, μm^2^	1 600‐10 600	2 200‐8 800
Median, maximum, μm^2^	9 200, 73 000	10 500, 133 000
Number of spectra	839	1 374

The data in ***X*** are of compositional nature because the relative values of ion counts are essential. An appropriate normalization or transformation has been performed by one of the two methods: (a) The normalization to a constant row sum of 100 describes the ions counts as percentages of the nine ion species and is preferred for interpretation. (b) A centered log‐ratio (CLR) transformation of ***X*** is recommended for compositional data,^13^ defined as *x*_CLR_[*i, j*] = log (*x*[*i, j*] /*G*[*i*]) with *G*[*i*] for the geometric mean of all variables of object *i.* Calculation of *G* requires values >0; to overcome this problem, x‐values lower than the 0.05 quantile (*q*_0.05_, separately for each variable) were replaced by uniformly distributed random numbers between *q*_0.05_/5 and *q*_0.05_.

### 2.2 Data evaluation methods

An exploratory data analysis^14^, ^15^, ^16^ for a visualization of the class discrimination has been performed by a robust PCA with CLR‐transformed data, and using the method *robPCA,* 17 as implemented in the function *PcaHubert()* from the R‐package *rrcov.* 18 This method yields orthogonal loading vectors, while the score vectors are in general not uncorrelated for the benefit of robustness.

For a discrimination of the two classes, three complementary methods have been used^16^: (a) linear discriminant analysis with partial least squares (DPLS) regression,^19^ (b) nonlinear *k*‐nearest neighbor (KNN) classification^20^ based on estimating local class densities, and (c) nonlinear random forest (RF) classification^21^, ^22^ based on thresholds for the variables in decision trees. The classification performance is expressed as the ratios of correctly assigned test set objects, separately for each class, the predictive abilities *P*_1_ and *P*_2._ 16 An appropriate single performance measure is *P*, the arithmetic mean of *P*_1_ and *P*_2_. The variation of these measures as obtained by repeated cross validation (rCV) (see below) is visualized by boxplots, and the means are used for a comparison of the methods in Table 2.

**Table 2:** Predictive abilities obtained by the applied classification methods DPLS, KNN, and RF

Method	Parameter	*P*_1_	*P*_2_	*P*
DPLS	*A*_OPT_ = 4	0.39	0.94	0.67
KNN	*k*_OPT_ = 1	0.73	0.87	0.80
RF	500 trees	0.76	0.91	0.83

*Note.* Means of 100 repetitions in repeated cross validation.

Abbreviations: DPLS: discriminant analysis with partial least squares; KNN: *k*‐nearest neighbor; RF: random forest.

Discriminant partial least squares regression has been applied in combination with repeated double cross validation, rdCV.^23^, ^24^ This strategy allows a separate estimation of the optimum number, *A*_OPT_, of PLS components and of the classification performance for test set objects, together with an estimation of the variabilities of these criteria for varying random splits into calibration and test sets. For PLS computation, the function *plsr()* from the R‐package *pls* has been applied.^25^ Autoscaled *x*‐data have been used because of a better model performance than with using sum 100 normalized data. The rdCV parameters were three segments in the outer CV loop (test set split), five segments in the inner CV loop (optimization of *A*, the number of PLS components), and 100 repetitions. The resulting optimum model complexity, *A*_OPT_, is the most frequent value obtained for *A.*

A final PLS model with *A*_OPT_ components using all objects has been calculated, and the resulting regression coefficients (standardized because of autoscaled *x*‐data) used as a measure for the variable importance.

*K*‐nearest neighbor classification was performed with sum 100 normalized *x*‐data, using the Euclidean distance and applying the function *knn()* from the R‐package *class.* KNN was combined with rdCV^24^ for a separate estimation of the optimum number of neighbors, *k*_OPT_, and of the model performance, together with their variabilities for varying random splits into calibration and test sets. The rdCV parameters were the same as used for DPLS.

RF classification is based on repeatedly created decision trees (the forest) applying the method CART (classification and regression trees) and using randomly selected subsets of the variables. RF calculations were done with the function *randomForest()* from the R‐package *randomForest,* 26 using sum 100 normalized *x*‐data. A rCV was applied with three segments (two for the training sets and one for the test sets), 100 repetitions, and 500 trees per training.

The function used for RF provides a measure for the variable importance for class separation, namely, the *mean decreasing accuracy* (MDA). MDA has a high value if the classification accuracy decreases considerably when the variable is eliminated. Variables with a high MDA can be considered as being relevant for the class separation.

Finally, the distributions of variable values and some ratios of variables have been presented by boxplots.^16^, ^27^ This robust visualization of distributions supports the interpretation of potential differences of the classes.

## 3 RESULTS

### 3.1 Exploration

**Figure 1:**
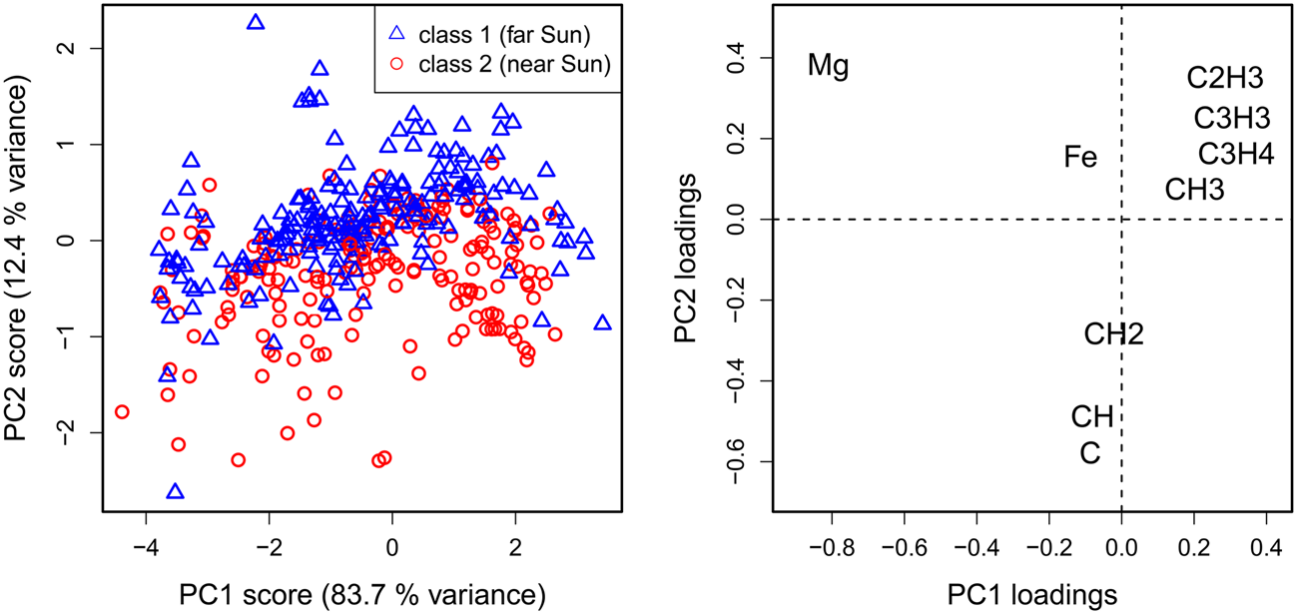
Robust principal component analysis (PCA) with a random sample of 200 spectra from each class; variables were centered log‐ratio‐transformed because of their compositional nature

A robust PCA was applied for a visualization of the data structure. Data sets with 200 randomly selected objects from each class have been used for a better readability of the score plots. Figure 1 shows a typical result with 83.7 and 12.4 % variance preserved by the first two components. The two classes exhibit a distinct separation; a complete separation cannot be expected as the definition of the classes implies an overlap and PCA does not consider any class information. The loading plot indicates a high relevance of the ions Mg^+^ and Fe^+^ for class 1 (sampling in the first period, far from Sun), and C^+^, CH^+^, and CH_2_^+^ for class 2 (sampling in the second period, near Sun).

**Figure 2:**
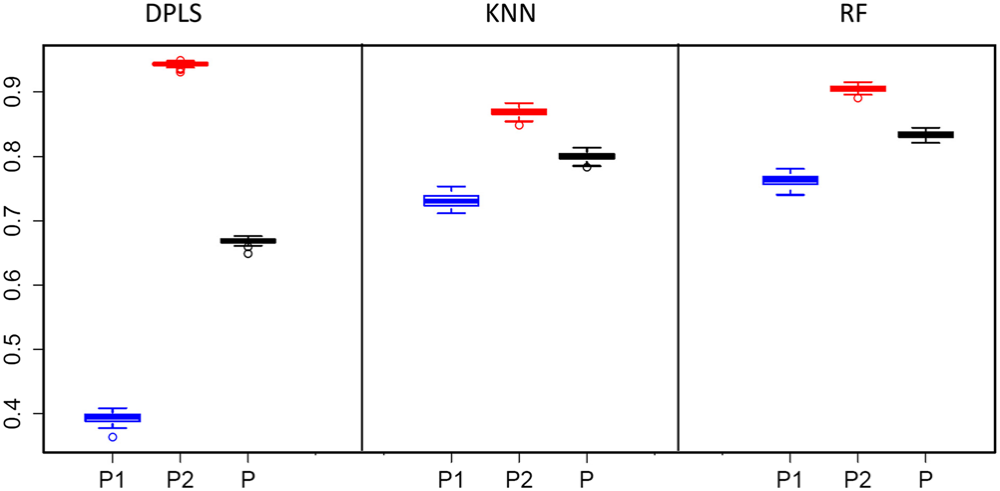
Variation of the predictive abilities of 100 repetitions in repeated cross validation. *P*_1_, samples from first period, far from the Sun; *P*_2_, samples from second period, near the Sun; *P* is the mean of both

### 3.2 Classification

The classification performances obtained by the applied three methods are summarized in Table 2. The linear method DPLS gives only a poor mean predictive ability, *P*, of 0.67; however, the nonlinear methods KNN and RF discriminate well with *P* = 0.80 and 0.83. The variability of the predictive abilities in rCV is small, due to the small number of variables, the high number of objects, and the obviously homogenous data (Figure 2). The distinct class discrimination based on mass spectral data indicates different particle compositions of the classes.

More specifically, the optimum number of neighbors, *k*_OPT_, in KNN is 1; however, the classification performance for increasing generalization considering up to 10 neighbors is stable. The RF classification shows only minor changes in the performance when varying the number of trees between 200 and 10 000.

### 3.3 Variable importance

The importance of the variables for class discrimination has been characterized by two approaches. (a) Univariate and bivariate methods allow a direct interpretation in terms of ion abundances and chemical composition. The class distributions of selected single variables and ratios of variables have been compared by boxplots. (b) For multivariate methods, the importance of a variable is influenced by the other variables. We focus on the MDA criterion from RF and compare it with the standardized regression coefficients from DPLS.

**Figure 3:**
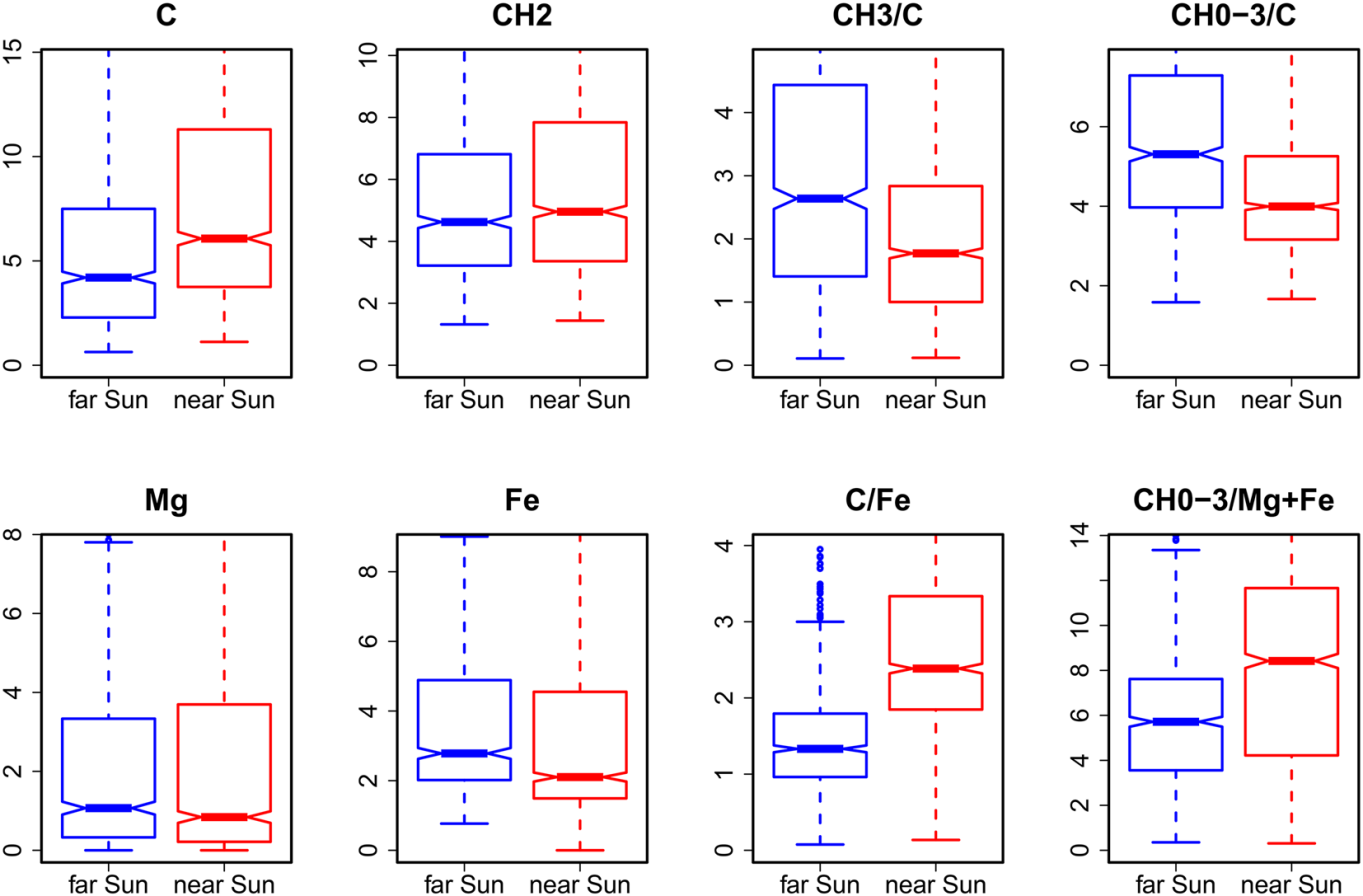
Boxplots showing the distributions of selected ion counts (sum 100 normalized) and ion count ratios for samples in class 1 (collected during the first period, far the Sun, left, in blue color) and class 2 (second period, near the Sun, right, red); values higher than the 0.9 quantile are cut

Single variables have highly overlapping distributions for class 1 and 2 as for example shown for the ions C^+^, CH_2_^+^, Mg^+^, and Fe^+^ in Figure 3. If the notches of two boxplots do not overlap, this is a strong evidence that the two medians differ. The ratios CH_3_^+^/C^+^ and CH_0‐3_^+^/C^+^ have significantly lower values in class 2 (second period, near Sun) than in class 1. CH_0‐3_^+^ stands for the sum of ion counts of C^+^, CH^+^, CH_2_^+^, and CH_3_^+^. Best discrimination give the ratios C^+^/Fe^+^ and CH_0‐3_^+^/(Mg^+^+Fe^+^). It is remarkable that carbon containing ions show increased abundances compared with Mg^+^ and Fe^+^ in spectra from samples collected in the second period rather near the Sun.

**Figure 4:**
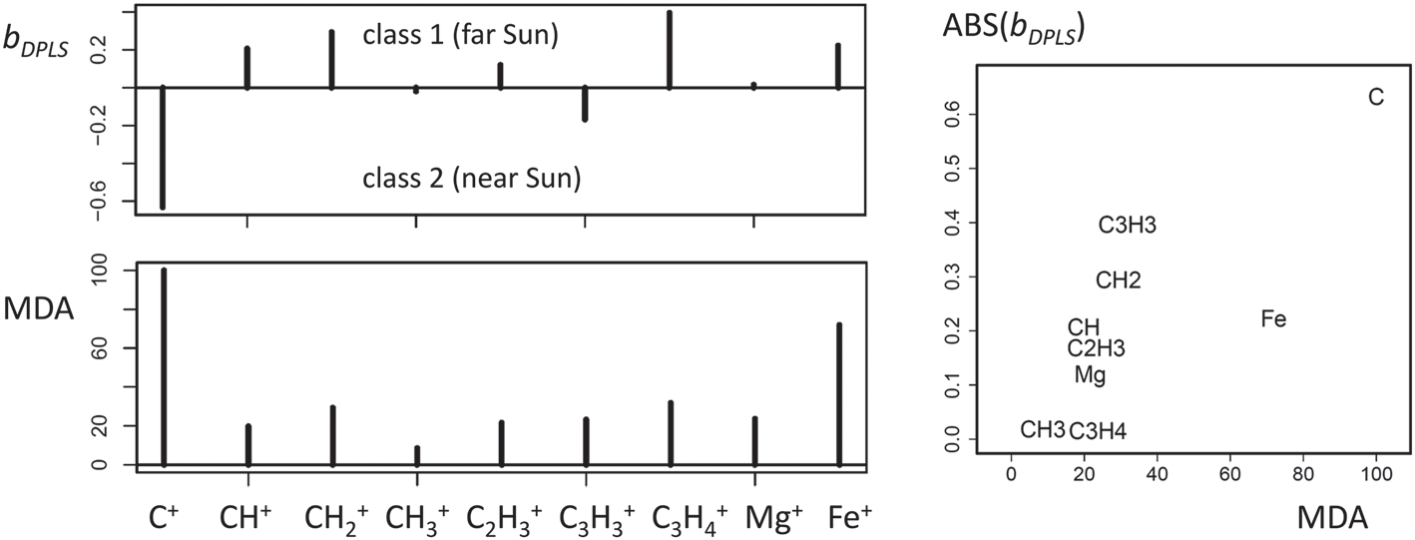
Importance of the variables for class separation with multivariate methods. *b*_DPLS_ standardized regression coefficient of a DPLS discriminant variable; MDA, mean decreasing accuracy from random forest classification

The variable importance obtained from the multivariate approaches RF and DPLS is shown in Figure 4. The MDA measure from RF discloses the relative abundances of C^+^ and Fe^+^ as most relevant for separating classes 1 and 2. The standardized regression coefficients, *b*_DPLS_, for a DPLS discriminant variable indicate that C^+^ is prominent in class 2 (near Sun), and especially C_3_H_4_^+^ and Fe^+^ are prominent in class 1; however, note that DPLS is less discriminating than RF or KNN (Table 2). MDA and absolute values of *b*_DPLS_ show similar trends with a squared Pearson correlation coefficient of 0.6.

## 4 SUMMARY AND DISCUSSION

Potential different compositions of two classes of cometary particles collected during two periods of the Rosetta mission have been investigated. The compositions are characterized by patterns of relative abundances of nine positive secondary ions measured by TOF‐SIMS. The selected ions characterize the organic and inorganic compositions of the samples. The applied multivariate methods (PCA, DPLS, KNN, and RF) show a separation of both classes and thus indicate different compositions. The nonlinear methods KNN and RF exhibit a much better class separation than the linear DPLS. Correspondingly, the class distributions for single variables show a high overlap; however, some ratios of ion abundances are well discriminating, for instance C^+^/Fe^+^ and CH_0‐3_^+^/(Mg^+^+Fe^+^). This data analysis gives clear evidence for enhanced relative ion abundances of C^+^ (and to a lesser extent of C_3_H_3_^+^) for samples collected in the second period (near the Sun, class 2). On the other hand, samples collected during the period between start of experiments (about 3.6 AU heliocentric distance) and before approaching a distance of 2.6 AU (class 1) have enhanced relative abundances of Fe^+^ together with smaller ratios C^+^/Fe^+^ than in class 2.

A discussion of these results in terms of comet science requires further investigations. The particles collected in the first part of the mission may be material from the dust coat of the comet that gets removed once the activity starts again (the orbital period is 6.44 years). This material is depleted in carbonaceous volatiles from former perihelion passes. As proposed by R. Schulz at al.,^28^ these particles may represent the parent material of interplanetary dust as contained in meteor streams with cometary origin. The fresh material emitted from the comet surface and collected at smaller heliocentric distances has definitely another composition with an enhanced content of carbonaceous matter.

## References

[1] Schulz R, Boehnhardt AC, Glassmeier KH (Eds). Rosetta: ESA's mission to the origin of the solar system. New York: Springer; 2009.

[2] Kissel J , Altwegg K , Clark BC , et al. Cosima –high resolution time‐of‐flight secondary ion mass spectrometer for the analysis of cometary dust particles onboard Rosetta. Space Science Reviews. 2007;128:823‐867.

[3] Langevin Y , Hilchenbach M , Ligier N , et al. Typology of dust particles collected by the COSIMA mass spectrometer in the inner coma of 67P/Churyumov Gerasimenko. Icarus. 2016;271:76‐97.

[4] Merouane S , Zaprudin B , Stenzel O , et al. Dust particle flux and size distribution in the coma of 67P/Churyumov‐Gerasimenko measured in situ by the COSIMA instrument on board Rosetta. Astron Astrophys. 2016;596:1‐12.A87.

[5] Hilchenbach M , Kissel J , Langevin Y , et al. Comet 67P/Churyumov–Gerasimenko: close‐up on dust particle fragments. The Astrophysical Journal Letters. 2016;816:1–6.L32.

[6] Fray N , Bardyn A , Cottin H , et al. High‐molecular‐weight organic matter in the particles of comet 67P/Churyumov‐Gerasimenko. Nature. 2016;528:72‐74. 10.1038/nature19320 27602514

[7] Bardyn A , Baklouti D , Cottin H , et al. Carbon‐rich dust in comet 67P/Churyumov‐Gerasimenko measured by COSIMA/Rosetta. MNRAS (Monthly Notices of the Royal Astronomical Society). 2017;469, supplement 2:S712‐S722.

[8] Isnard R , Bardyn A , Fray N , et al. H/C elemental ratio of the refractory organic matter in cometary particles of 67P/Churyumov‐Gerasimenko. Astronomy Astrophysics. 2019;630:1–10.A27.

[9] Fray N , Bardyn A , Cottin H , et al. Nitrogen‐to‐carbon atomic ratio measured by COSIMA in the particles of comet 67P/Churyumov‐Gerasimenko. MNRAS (Monthly Notices of the Royal Astronomical Society). 2017;469:S506‐S516.

[10] Varmuza K , Filzmoser P , Hoffmann I , et al. Significance of variables for discrimination: applied to the search of organic ions in mass spectra measured on cometary particles. J Chemometr. 2018;32:1–13.e3001.

[11] Stenzel O , Hilchenbach M , Merouane S , et al. Similarities in element content between comet 67P/Churyumov‐Gerasimenko coma dust and selected meteorite samples. MNRAS (Monthly Notices of the Royal Astronomical Society). 2017;469, supplement 2:S492‐S505.

[12] Merouane S , Stenzel O , Hilchenbach M , et al. Evolution of the physical properties of dust and cometary dust activity from 67P/Churyumov‐Gerasimenko measured in situ by Rosetta/COSIMA. MNRAS (Monthly Notices of the Royal Astronomical Society). 2017;469:S459‐S474.

[13] Filzmoser P , Hron K , Templ M . Applied compositional data analysis. Springer Nature Switzerland: Cham (Switzerland); 2018.

[14] Brereton RG . Applied chemometrics for scientists. Chichester, United Kingdom: Wiley; 2007.

[15] Manly BFJ . Multivariate statistical methods: a primer. London, United Kingdom: Chapman and Hall; 2000.

[16] Varmuza K , Filzmoser P . Introduction to multivariate statistical analysis in chemometrics. Boca Raton, FL, USA: CRC Press; 2009.

[17] Hubert M , Rousseeuw PJ , VandenBranden K . ROBPCA: a new approach to robust principal components. Technometrics. 2005;47:64‐79.

[18] Todorov V , Filzmoser P . Robust multivariate analysis. J Stat Softw. 2009;32:1‐47.

[19] Brereton RG , Lloyd GR . Partial least squares discriminant analysis: taking the magic away. J Chemometr. 2013;28:213‐225.

[20] Brereton RG . Chemometrics for pattern recognition. Chichester, United Kingdom: Wiley; 2009.

[21] Breiman L . Random forests. Machine learning. 2001;45:5‐32.

[22] Breiman L , Friedman J , Stone CJ , Olshen RA . Classification and regression trees. New York, USA: Chapman & Hall; 1984.

[23] Filzmoser P , Liebmann B , Varmuza K . Repeated double cross validation. J Chemometr. 2009;23:160‐171.

[24] Varmuza K , Filzmoser P . Repeated double cross validation (rdCV)—a strategy for optimizing empirical multivariate models, and for comparing their prediction performances In: Khanmohammadi M, ed. Current applications of chemometrics. New York, NY, USA: Nova Science Publishers; 2015:15‐31.

[25] Wehrens R . Chemometrics with R. Heidelberg, Germany: Springer; 2011.

[26] Liaw A , Wiener M . Classification and regression by random forest. R News. 2002;2:18‐22.

[27] Venables WN , Ripley BD . Modern applied statistics with S. New York, NY, USA: Springer; 2003.

[28] Schulz R , Hilchenbach M , Langevin Y , et al. Comet 67P/Churyumov‐Gerasimenko sheds dust coat accumulated over the past four years. Nature. 2015;518:216‐218. 25624103 10.1038/nature14159

